# Clinical Indications and Outcomes of Sinus Floor Augmentation With Bone Substitutes: An Evidence‐Based Review

**DOI:** 10.1111/cid.13400

**Published:** 2024-10-17

**Authors:** Muhammad H. A. Saleh, Hamoun Sabri, Natalia Di Pietro, Luca Comuzzi, Nicolas C. Geurs, Layal Bou Semaan, Adriano Piattelli

**Affiliations:** ^1^ Department of Periodontics and Oral Medicine University of Michigan School of Dentistry Ann Arbor Michigan USA; ^2^ Department of Medical, Oral and Biotechnological Sciences “G. d'Annunzio” University of Chieti‐Pescara Chieti Italy; ^3^ Center for Advanced Studies and Technologies (CAST) “G. d'Annunzio” University of Chieti‐Pescara Chieti Italy; ^4^ Independent Researcher San Vendemiano‐Conegliano Veneto Treviso Italy; ^5^ School of Dentistry, Department of Periodontology University of Alabama at Birmingham Birmingham Alabama USA; ^6^ School of Dentistry Saint Camillus International University of Health and Medical Sciences (UniCamillus) Rome Italy; ^7^ Facultad de Medicina UCAM Universidad Católica San Antonio de Murcia Murcia Spain

**Keywords:** bone augmentation, dental implants, maxillary sinus, sinus augmentation, sinus floor elevation, sinus lift

## 1 Introduction

Tooth loss, resulting from various causes such as periodontal disease or dental caries, can lead to the subsequent resorption of the alveolar bone process [1]. This fundamentally complicates implant‐based rehabilitation, especially in the posterior maxilla. Crestal migration of the maxillary sinus floor due to pneumatization, combined with resorption of the alveolar bone, can result in inadequate residual bone height. This situation may require pre‐implant interventions like sinus floor elevation (SFE) to ensure enough bone for implant placement [2, 3].

Several strategies have been introduced to increase the bone height in the posterior maxilla [3, 4, 5]. SFE has been shown to have relatively high success in augmenting the posterior maxilla with a deficient bone height. Two main techniques have been introduced: (1) The transalveolar (vertical, closed) SFE, and (2) The lateral window approach (open). It should be noted that several modifications of these techniques have also been introduced [6, 7, 8].

Given that this regenerative intervention aims to enhance bone height and width to facilitate proper implant placement, dental implants can be placed simultaneously with the sinus augmentation procedure (referred to as the “one‐stage” technique). Alternatively, a staged approach may be employed, where bone augmentation occurs during the initial surgical intervention, followed by the placement of dental implants once adequate bone volume has been established (known as the “two‐stage” technique) [9, 10].

The classical sinus lift procedure, introduced by Tatum in the 1970s, involves a lateral window approach [11, 12]. This technique involves creating incisions to expose the sinus wall, followed by a trapdoor osteotomy to access the sinus membrane and cavity. Careful dissection and membrane elevation are performed to create space for graft material. If sufficient basal bone is present, implants may be placed simultaneously, protruding through the sinus cavity and protected by the sinus membrane. The remaining space is typically filled with bone replacement grafts, and the window opening is closed with a barrier membrane. However, a two‐stage approach is required if the basal bone is inadequate, and implants are placed only after sufficient bone regeneration [9].

In contrast, an alternative, less‐invasive technique introduced by Tatum and modified by Summers is the crestal approach [13]. When indicated, this approach involves elevating the sinus floor through the alveolar crest using osteotomes. Summers' modification utilizes concave‐tipped tapered osteotomes to fracture the maxillary floor and elevate the sinus membrane. This technique is less invasive, less time‐consuming, and allows for better bone density and implant stability due to lateral compression exerted by the osteotomes. After membrane elevation, various bone grafting materials may be used to fill the resulting space. However, the need for graft material post‐lifting has been debated, as clot stabilization may promote new bone formation [14]. Despite favorable implant survival rates exceeding 90% reported in systematic reviews, blind procedures carry risks of complications [15].

### 1.1 Aim

This article aims to comprehensively explore different bone grafting materials, histologic, and clinical results, and clinical recommendations for using biomaterials during SFE. The concept of graft‐free SFE will be discussed, various grafting materials will be compared, and their roles in influencing biological processes will be evaluated. Additionally, histologic and clinical results will be examined to assess their correlation with clinical outcomes. Clinical recommendations for biomaterial selection during SFE will be provided, and this manuscript will offer suggestions for future research.

## 2 Different Grafting Materials and Respective Clinical and Histological Outcomes

### 2.1 Graft‐Free Sinus Floor Elevation

Several limitations regarding bone graft substitutes in maxillary SFE procedures, such as increased costs, need for autogenous bone harvesting, increased surgical time, increased chance of immunogenic response (in non‐autogenous grafts), and complications further necessitated the concept of graft‐free (also known as coagulum, blood‐only or graftless) SFE [16]. The biological foundation of this approach was derived from the concept of new bone formation following the shrinkage and ossification of the blood clot. This was investigated in a study by Palma et al. [17] on four tufted capuchin primates, where they conducted a comparative histological analysis of sinus membrane elevation using the lateral window technique and simultaneous implant placement with and without adjunctive autogenous bone graft. They found that the sinus floor provided approximately 2.2 mm (SD ± 1.1 mm) of cortical bone for primary stability, while the remaining implant projected into the sinus cavity, which subsequently filled with new bone over time. Notably, the histological examination revealed that sites with membrane elevation exhibited the highest new bone formation at the periphery, often extending toward the center of the augmented area.

In contrast, grafted sites showed less bone tissue lining the sinus membrane at the uppermost part of the implant. Moreover, the study investigated different patterns of implant integration based on surface modification. For instance, implants with an oxidized surface demonstrated direct bone formation without evidence of trabeculae projection from the surroundings. In contrast, those with a machined surface showed bone growth primarily from the periphery onto the implants. Morphometric measurements indicated a markedly higher degree of bone‐implant contact (BIC) for oxidized implants than machined implants. In a split‐mouth pilot randomized controlled trial (RCT), Lie et al. [18] compared graftless lateral SFE with a mixture of autogenous and xenogenous bone grafts. The results of the no‐graft group revealed significant new bone formation in all human bone specimens. Histological evaluations demonstrated a smooth transition zone between residual and newly formed bone by osteoinductivity. This indicates spontaneous bone regeneration in the absence of grafting materials. The lack of inflammatory reaction in the neighborhood of the augmented site suggests a favorable environment for bone healing without the need for additional grafting materials. This was in accordance with an animal study by Cricchio et al. [19] on six tufted capuchin primates, where a space‐making device was utilized to facilitate two‐stage lateral SFE. Histological examination revealed consistent bone formation in contact with the Schneiderian membrane and the space‐making device, even when displaced. The study highlighted the importance of the space‐making device's stability for predictable bone augmentation to ensure a sustained connection between the membrane and the secluded space.

Shifting from the histological and biological outcomes toward clinical results of graft‐free SFEs, several parallel [20, 21, 22, 23, 24] and split‐mouth design RCTs can be noted when lateral window SFE is performed. Fouad et al. [24] performed 20 lateral SFE (with 34 simultaneous implants) on sites with 4–6 mm residual bone height using either a xenograft or graftless approach. The 6‐month results revealed a significantly lower bone height gain for the graftless approach (8.59 ± 0.74 mm vs. 4.85 ± 0.5 mm). In a 2‐year follow‐up study, Ranaan et al. [23] performed a graftless approach following the Slit‐window graft‐free SFE technique where a rigid bovine pericardium membrane (CopiOs Pericardium Membrane, Zimmer Dental) was inserted within two slits to creating a “tented area” that stabilized the Schneiderian membrane in the elevated position. In comparison to the control group (corticocancellous particulate allograft (Puros, Zimmer Dental) and a pericardium membrane [CopiOs Pericardium Membrane, Zimmer Dental]), a significantly lower bone height was achieved (11.6 mm vs. 13.05 mm); mean difference −1.79 mm (95% confidence intervals CIs: −3.33, −0.26; *p* = 0.024). Furthermore, a recent systematic review with meta‐analysis by Lie et al. [6] summarized nine RCTs on lateral SFE with at least one arm of the graftless approach. Briefly, their results indicated a high overall implant survival rate in both the graftless and grafted SFE groups (97.92% and 98.73%, respectively). The graftless group showed a significantly lower vertical bone height gain, with a mean difference of −1.73 mm (*p* = 0.01), and a significantly lower bone density, with a mean difference of −94.7 HU (*p* < 0.001). Conversely, the implant stability quotient (ISQ) levels did not differ between the two groups (*p* = 0.07). In a more recent systematic review with trial sequential analysis by Chen et al. [25], with inclusion of higher number of RCTs (*n* = 17) comparing graft versus graftless approaches, no statistically significant difference between the two groups in terms of the implant survival rate in the short term (*n* = 11, *I* ^2^ = 0%, risk difference (RD): 0.03, 95% (CI): −0.01 to −0.07, *p* = 0.17, required information size (RIS) = 307). Although the grafting approach had a higher endo‐sinus bone gain (*n* = 13, *I* ^2^ = 89%, weighted mean difference (WMD): –1.24, 95% CI: −1.91 to −0.57, *p* = 0.0003, RIS = 461), this was not a determining factor in implant survival. No difference in perforation (*n* = 13, *I* ^2^ = 0%, RD = 0.03, 95% CI: −0.02 to −0.09, *p* = 0.99, RIS = 223) and marginal bone loss (*n* = 4, *I* ^2^ = 0%, WMD = 0.05, 95% CI: −0.14 to −0.23, *p* = 0.62, no RIS) was detected between the two groups using meta‐analysis.

Despite the mentioned successful outcomes, the limitations of using graftless SFE should also be acknowledged. An intact Schneiderian membrane is a crucial prerequisite of this approach. Considering the high incidence of membrane perforations in lateral SFE [26], this technique may be inapplicable. Moreover, some current clinical studies indicate that in extremely resorbed ridges (less than 4 mm), grafting approaches would result in more new bone formation compared to coagulum only.

#### 2.1.1 Clinical Recommendations

It has been observed that bone neoformation and implant osseointegration after SFE without grafting material is possible. It remains necessary to acknowledge the indications and the contraindications of the sole use of the blood clot in maxillary sinuses for bone regeneration. The size of the defect, when it comes to the extent and the amount of the residual bone height, has a significant impact on the formation of intra‐sinus bone. Major extensions or increased fills of the maxillary sinus are not viable solely with a blood clot and may need additional graft material [27]. Reduced treatment time, decreased percentage of remaining graft particles, reduced number of complications such as infection, and lower treatment costs are possible advantages of the graft‐free SFE [28, 29].

### 2.2 Autologous Bone

This material remains the gold standard in bone regeneration procedures. Its predominant limitations are limited clinical availability and the possibility of complications, morbidity, and possible pain when retrieved from oral (e.g., symphysis, ramus) or extraoral sites (e.g., iliac crest). The grafted particles of autologous bone have a histological aspect very similar to that of the host bone, with most particles surrounded by newly formed bone. In studies where high power fields (HPF) were used [26, 27], an active deposition of osteoid matrix by osteoblasts directly on the surface of the grafted material was observed, with osteoclasts and macrophages typically absent. Newly formed bone, distinguished by its higher affinity for dyes, was separated from pre‐existing bone by a structure resembling a cement line. Furthermore, it was reported that cortical autologous bone undergoes slow resorption while medullary bone experiences significantly higher resorption. In summary, the autologous bone is highly biocompatible, osteoinductive, and osteoconductive, with the cortical portion demonstrating a notably slower resorption rate than the cancellous.

Composite grafting protocols have been developed to overcome the limited availability and unpredictable resorption of autogenous bone. A recent RCT by Starch‐Jensen et al. [30], performed 60 lateral window SFE with simultaneous implant placement (control: autogenous bone from zygomatic buttress; Test 1: 1:1 autogenous + anorganic porcine bone mineral; Test 2: 1:1 autogenous + biphasic bone graft material). After 1 year of function, neither of the clinical and patient‐reported outcomes significantly differed among the groups, suggesting comparable results of the test group versus autogenous bone alone. These results, supported by earlier studies [28, 29, 31], indicate that extensive autogenous bone harvesting for SFEs may not be necessary.

Autogenous bone grafting continues to present with consistent reliability, though resorption of these grafts can occur. In a study by Jamcoski et al. [30], they observed a 97% implant success rate when placed in autogenous bone grafts over a 15‐year period, which aligns closely with the results reported by Martuscelli et al. [32], who achieved a 100% success rate in a smaller cohort over a five‐year span.

Despite a few clinical studies on the use of autogenous dentin particulate graft as a graft material for SFE, data on the feasibility of the graft still needs to be made available. Therefore, no recommendations can be made at this stage [33].

#### 2.2.1 Clinical Recommendations

Grafting with autogenous bone is still a valid option for SFE thanks to its predictability and capacity to promote the activation and release of growth factors. However, these grafts can also be highly reabsorbed, whereas the reabsorption rate of the organic or synthetic bone grafting materials is comparatively lower.

Regardless of the high implant success rates associated with the autogenous bone, due to the associated morbidity and postoperative complications at the donor sites, clinicians may opt to avoid the secondary surgical site for bone harvesting. Reducing the volume of autogenous grafts by combining with bone substitutes does not compromise the success of implants placed in SFE sites. Combining autogenous bone with bone substitutes may be a more suitable option [30, 34].

### 2.3 Anorganic Bovine Bone

Anorganic bovine bone (ABB) is a biomaterial (Bio‐Oss, Geistlich, Wohlhusen, Switzerland), from which the organic matrix has been completely removed, leaving only the mineralized portion [28, 35]. The material's porosity is 75%–80%, and the crystal dimensions are about 10 μm. The studies have shown that most of the grafted particles appear to be lined by newly formed bone that is always in close contact with the biappearsial surface, with no gaps nor the presence of fibrous, connective tissue at the interface; in some other areas, the newly formed bone appears to have bridged some particles [36, 38]. The histomorphometric evidence from these studies indicates that well‐organized osteons are present near some of the grafted biomaterial particles and the newly formed bone [36, 37, 38]. In most of the particles, the Haversian canals are colonized by cells and capillaries; in the internal portions of some of these canals, it was possible to observe an incomplete mineralized material [33]. In certain HPF, a perimeter of osteoblasts was evident, actively depositing osteoid matrix that underwent mineralization directly on the surface of the biomaterial [39]. More importantly, no signs of inflammatory cell infiltration or foreign body reaction cells were detected.

Moreover, the newly formed bone was easily distinguished from the grafted particles and the pre‐existing bone due to its higher affinity for dyes [36]. After being placed in sinuses augmented with ABB, a consistent pattern was observed where implants were retrieved to observe the interfaces between the implant surface, biomaterials grafted particles, and newly formed bone. A thin layer of newly‐formed bone was always interposed between the grafted particles and the implant surface, but no direct contact was found between the particles and the titanium [40, 41]. Most grafted particles contained cells within their osteocyte lacunae, while in a few instances, broad osteocyte lacunae were closely juxtaposed to the particles' external surface. In very few HPF, it was possible to observe osteoclasts, especially around particles not in contact with bone. These particles appeared to undergo a very slow resorption, and it was possible to observe their presence in the regenerated tissues even after several years after insertion in the host tissues. In a 2005 study by Orsini et al. [27], in some specimens evaluated under scanning electron microscopy (SEM), newly formed bone was observed protruding into the ABB particles in SFE cases. Moreover, in a 9‐year follow‐up study after SFEs [42] the residual grafted particles were also found in specimens retrieved from humans. Still, they had not interfered with the development of new osseous tissue and had not caused detrimental or unfavorable consequences. A later study by the same group [43] evaluated the histological results of human cores retrieved 6 months after SFEs, using different types of bone substitute biomaterials, indicating that the newly regenerated bone presented a structure similar to a D3 bone type, while only in a specimen of ABB, retrieved after a much more extended period of time, the osseous tissue presented features corresponding to D2 bone type. Consequently, we may infer from that increased bone density may occur over longer periods. This biomaterial exhibits exceptional biocompatibility, outstanding osteoconductivity, and remarkably slow resorbability.

In a study by Ferreira et al. [44], evaluating the use of ABB with a collagen membrane (CM) for sinus grafting in cases with residual bone height ≤ 7 mm of bone, 406 sinuses in 314 patients were treated, resulting in the placement of 1025 implants. Among these, 118 implants were placed in a one‐stage procedure, while 907 were placed 6–12 months later in a two‐stage approach. Histomorphometric analysis showed 39.0% new bone formation, 52.9% marrow space, and 8% residual ABB. The study concluded that ABB combined with CM provides a highly predictable implant survival rate of 98.1%.

In attempting to enhance the healing capacity of ABBs, several trials also tested additional benefits of autologous platelet concentrates (APCs) to enhance the healing quality of SFEs using ABB. In regenerative dentistry, APCs are biological products derived from the patient's blood and are used to promote healing and tissue regeneration. They are rich in growth factors, which are crucial in wound healing, tissue regeneration, and the stimulation of stem cells. Several RCTs have tested the benefits of APCs when added to DBBM to enhance healing outcomes [40, 45, 46, 47, 48, 49]. These mainly include the application of platelet‐rich plasma (PRP), plasma rich in growth factors (PRGF), and platelet‐rich fibrin (PRF). Likewise, the addition of bone marrow aspirate concentrates (BMACs) as well as EMD to DBBM (Bio‐Oss) has been shown to improve the new bone formation compared to Bio‐Oss alone [41, 50]. The overall trend shows improved patient‐reported outcomes (reduced pain/discomfort), as well as faster new bone formation based on biopsies taken. Nevertheless, in the long term, the limited number of these studies indicates higher amounts of new bone formation. Overall, the addition of these agents to bone grafts would result in improved healing outcomes as well as a possible reduction in the volume of bone used for grafting.

#### 2.3.1 Clinical Recommendations

ABB demonstrates biocompatibility, osteoconductivity and supports the growth of new bone tissue onto its surface. It exhibits remarkably slow resorption rates and can provide long‐term structural support with a gradual replacement by newly formed bone. When ABB is used in procedures such as SFE alongside implants, a thin layer of newly formed bone is observed between the grafted particles and the implant surface. This interface supports osseointegration and helps maintain stability. Additionally, it can be further optimized by combining it with biologics such as autologous platelet concentrates or bone marrow aspirate concentrates to enhance healing outcomes [51].

### 2.4 Porcine Bone

In porcine bone material (OsteoBiol by Tecnoss, Giaveno, Torino, Italy), the original and cutting‐edge production process allows for maintaining the presence and structure of Collagen type I and the characteristics of HA granules, avoiding the ceramization of the material [52]. Several studies have demonstrated conclusively that collagen plays a key role in accelerating new blood vessel formation, besides assisting in the differentiation of the stem cells present in the bone marrow, in the attachment of the endothelial cells, and in the agglomeration of the platelets [53, 54, 55]. This increase in the formation of small new blood vessels was, in all probability, due to an improved production of vascular endothelial growth factor (VEGF) in the presence of this collagenated biomaterial [26].

From a histological point of view, almost all the particles were surrounded by newly formed bone [56, 57]. At higher magnification, this bone was found to be in close contact with the grafted particles' surface, with no gaps or fibrous connective tissue at the interface. No inflammatory infiltration or foreign body reaction cells were ever found in all the specimens. Moreover, some grafted particles appeared to be bridged by the newly formed bone and wide osteocyte lacunae were observed in the newly formed bone. The grafted biomaterial particles, exhibiting reduced affinity, were readily discernible from the newly formed bone, with most displaying colonization of Haversian canals by cells and capillaries, along with the presence of a fuchsine acid‐positive substance observed in a select few Haversian canals. Also, osteocyte lacunae located in the most peripheral particles appeared to be, almost in all samples, filled by osteocytes, while, in the most central particles, cells with morphologies different from that of osteocytes were found in the lacunae. In the reviewed studies, only in a limited number of cases, osteocyte lacunae appeared to be empty.

Porcine bone biomaterials exist in different formulations with different clinical indications:

*OsteoBiol Apatos*: Composed by cortico‐cancellous or cortical bone with a microcrystalline structure, this biomaterial is a versatile filler for treating peri‐implant and two‐wall defects, fitting well in large sockets post‐molar extractions, and suitable for SFEs (crestal or lateral window), horizontal regenerations, and combined with evolution membrane for superior ridge preservation compared to non‐preserved sites [58].

*OsteoBiol Gen‐Os*: Constituted by a cortico‐cancellous bone blend (100% collagenated bone blend). It is a bone substitute biomaterial composed of dehydrated granules. Each granule contains about 22% collagen embedded in the organic‐mineral matrix. This biomaterial can be used in every indication in bone regeneration procedures. *OsteoBiol Gen‐Os* was investigated in various surgical protocols, including SFE. In an RCT on twenty‐four patients, 9 months after mini‐implants placement, Hirota et al. [59] reported an amount of newly formed bone in contact with the implant surface between 40.9% ± 11.9% and 48.5% ± 20.1% depending on the position of the antrostomy. Another clinical study performed by Imai and collaborators evaluated the BIC, 9 months after the placement of mini‐implants. They found a BIC of 41.1% ± 19.5% and 42.8% ± 13.2% for large and small antrostomy, respectively. Furthermore, the authors reported a scarce number of dual‐phase biomaterials in contact with the implant surface. When used in vertical augmentation procedures with the shell technique, it needs to be mixed with 50% autologous bone [60].

*OsteoBiol mp3*: A combination of 90% of 600–1000 μm collagenated granules with 10% collagen gel, it is prehydrated in a syringe and can be successfully used in lateral SFE procedures and in post‐extraction sockets [54]. A split‐mouth RCT presented similar results between sinuses elevated either with autogenous bone chips or OsteoBiol mp3. Howship lacunae and osteoblasts detected on histological samples taken 6 months after the grafting of the dual‐phase biomaterial of porcine origin witness an ongoing remodeling process [61]. In addition, comparable results in terms of marginal bone loss and implant survival rate were reported over a period of three years [62].

*OsteoBiol GTO*: Identical composition and size of the granules to that of OsteoBiol *mp3*, though, with an addition of a 20% TSV Gel (i.e., a collagen gel enriched with a thermosensitive copolymer that renders the final product sticky). It is provided in syringes, and it can be successfully used in sockets that have lost the vestibular cortex, peri‐implant dehiscence, horizontal regeneration of knife‐edge alveolar crests and SFEs [55], and lateral maxillary sinus augmentation [63].

*OsteoBiol Gel 40*: A composition of a collagenated pre‐hydrated bone mixture with granulometry ≤300 μm with a mixture of 60% collagenated bone composition with 40% collagen gel. It can be used successfully for crestal SFEs with immediate implantation [64]. The 40% collagen gel allows an unproblematic detachment of the Schneider membrane, as reported by Lombardi et al. Moreover, out of 71 implants placed, 54 were inserted simultaneously into the sinus membrane elevation [65]. It should be noted that the 40% collagen gel resorbs faster than bone granules, and, for this reason, a predictable shrinkage of about 36% should be expected when using OsteoBiol Gel 40. The shrinkage rate of different biomaterials was investigated by Comuzzi et al. [66], who reported bigger vertical and mesiodistal changes for bone granules with a diameter lower than 300 μm.

*OsteoBiol Putty*: An identical composition as Gel 40 with a ratio micro‐granules/collagen gel 80/20. It is a biomaterial ideal for treating self‐containing peri‐implant defects, but it can also be used in crestal sinus augmentation procedures [66] with immediate implantation [64].

All the mentioned biomaterials exhibit exceptional biocompatibility and high osteoconductivity due to their extensive interconnected pores. Resorption rates vary between formulations; for instance, OsteoBiol mp3 typically undergoes resorption within approximately 8 weeks in a rabbit study [53].

All these different types of porcine biomaterials have been found capable of promoting tissue regeneration, thanks to their ability to enhance cell growth and differentiation. Furthermore, using transmission electron microscopy (TEM), it was possible to observe all stages of bone formation (i.e., osteoid matrix, trabecular bone, and lamellar bone). Interestingly, the residual implanted particles did not hinder the formation of new bone tissue, nor did they induce negative or harmful outcomes.

A randomized controlled clinical trial aimed to compare marginal bone loss using a split‐mouth design after bilateral sinus floor augmentation, with one side receiving autologous bone from the mandible and the other porcine xenograft. After 6 months, a total of 39 dental implants were placed in the posterior maxilla, with both graft materials showing high implant survival rates at 12 months—95% for the xenograft side and 100% for the autologous side [67]. Similar results were found by Forabosco et al., when evaluating the impact of concentrated growth factors matrix on implant survival rates and postoperative morbidity in maxillary sinus augmentation in fifty cases using OsteoBiol. After 1 year, implant survival rates were 96.4% for the group grafted with OsteoBiol mixed with concentrated growth factors matrix and 96.1% for the control group grafted only with OsteoBiol, with no significant difference between them [68].

#### 2.4.1 Clinical Recommendations

Porcine bone substitutes demonstrate several advantageous properties for clinical use, including enhanced angiogenesis and excellent histological integration. These products have a unique production process that retains the structure of collagen type i and hydroxyapatite (HA) granules. The presence of collagen is crucial for enhancing new blood vessel formation and supporting cellular activities essential for bone healing and integration. Histological studies confirm that these biomaterials integrate well with newly formed bone, without inflammatory reactions or fibrous tissue formation, making it a valuable biomaterial for a variety of bone grafting applications, including SFE procedures.

### 2.5 Demineralized Freeze‐Dried Bone Allograft

This biomaterial (Life‐Net Services, Norfolk, VA, USA) has the characteristic of being a very slowly resorbable biomaterial, and it has been reported to take significant time to be resorbed and substituted by newly‐formed bone [69]. The grafted particles located near the pre‐existing bone were, for the most part, lined by newly formed bone and appeared to be undergoing a partial demineralization, with areas positive to basic fuchsine and areas positive to silver nitrate (Von Kossa) [70, 71]. In these most peripheral particles, remineralization nuclei, positive to acid fuchsine, were found inside the grafted particles. These nuclei tended to fuse until there was a complete remineralization of the demineralized matrix. However, the remineralization of the grafted particles was neither homogeneous nor complete, and it often failed to conform to the original configuration of the grafted particles [69]. Areas of mineralization were also found at the interface between grafted particles and newly formed bone. Only in some areas, the grafted particles were surrounded by newly formed bone. Fibroblasts, connective tissue, and capillaries were found in other areas. A few macrophages were also present. Some particles were found immersed in connective tissue and were not undergoing any mineralization processes. They presented an irregular characteristic with weak staining. Newly formed bone trabeculae showed different histological aspects, some having features of mature, compact, lamellar bone with osteons, while, in other areas, dystrophic mineralized tissue was present, similar to interglobular dentin. In only a few areas, it was possible to observe inflammatory infiltration. This biomaterial exhibits slow resorption and remineralization, along with low osteoconductivity.

When compared with xenograft, the results showed no significant difference in implant survival rates between the two groups (94.0% for demineralized freeze‐dried bone allograft (DFDBA) vs. 94.4% for xenografts) in a retrospective analysis of 107 patients requiring maxillary bone reconstruction where 141 sinuses were grafted, and 191 implants were placed. Complications occurred in 19.6% of patients, with a higher, though not statistically significant, incidence in the allograft group [72].

#### 2.5.1 Clinical Recommendations

DFDBA is suitable for treating various bone defects, including SFE, where slow resorption and long‐term stability are desired. Due to its low osteoconductivity, mixing with more osteoconductive materials, such as synthetic bone substitutes, could be considered. Patients should be informed about the remodeling timeline, setting realistic expectations regarding the healing process and the potential need for extended treatment.

### 2.6 Mineralized Freeze‐Dried Bone Allograft

The histological evidence on this material shows that the grafted biomaterial particles (Miami Tissue Bank, Miami, FL, USA) located near the pre‐existing bone appeared to be completely surrounded by newly‐formed bone [70]. Moreover, this newly formed bone was highly mineralized and had wide osteocyte lacunae. Contrary to this, grafted biomaterial particles situated distantly from pre‐existing bone were typically enveloped by a thin layer of newly formed bone, with some containing cells within osteocyte lacunae. Inflammatory infiltrate was consistently absent, while in selected HPFs, osteoblasts were seen depositing osteoid matrix directly on particle surfaces. Additionally, within certain grafted particles, osteons with Haversian canals containing capillaries and connective tissue were observed, along with the occasional presence of osteoclasts on particle surfaces. The biomaterials exhibited osteoconductive properties and were slowly resorbable. One of the most important aspects of bone regeneration procedures is the possibility of early vascularization of the grafted biomaterial particles. Chavarri‐Prado et al. [56], in a recently published study, compared with BBM and mineralized freeze‐dried bone allograft (FDBA) and found a higher percentage of bone formation using FDBA. Chae et al. [57], in a rabbit calvarial defects study, reported that decalcified human tooth bone improved bone formation when compared with undecalcified human tooth bone and anorganic bovine bone, after an 8‐weeks healing period.

#### 2.6.1 Clinical Recommendations

FDBA is slowly resorbable, which suggests it provides a stable scaffold for bone formation over an extended period, which is beneficial for the long‐term stability of the grafted area. The presence of osteoblasts depositing osteoid matrix directly on FDBA particles, and the observation of osteons with Haversian canals within the grafted particles, indicate that FDBA supports active bone remodeling and regeneration. It is associated with an absence of inflammatory infiltration, making it a safe choice with minimal risk of adverse immune responses.

### 2.7 Bone Morphogenic Proteins (See Supporting Information)

#### 2.7.1 Clinical Recommendations

While several studies have shown clinical efficacy of rhBMP‐2 (as rhBMP‐2/HA, rhBMP‐2/AC, or in combination with other bone graft materials), clinicians should weigh the cost‐effectiveness of using this material. Also, despite the mentioned clinical outcomes, patient‐centered outcomes are still scarce. Having mentioned that, possible complications (such as infection or swelling) should also be acknowledged. In summary, this material, where bone grafting materials are not applicable for any reason, may serve as a valuable alternative for SFEs [73].

### 2.8 Calcium Carbonate (See Supporting Information)

#### 2.8.1 Clinical Recommendations

Calcium carbonate is suitable for use in SFEs, demonstrating effective bridging of newly formed bone with the biomaterial particles, no gaps or connective tissue at the interface, and significant vertical bone gain. It has high osteoconductivity and biocompatibility, but it is also inherent with significantly great resorbability. It still can be used as a sole material. It is recommended to combine it with biologic/autologous agents to enhance healing outcomes. Further studies need to demonstrate the efficiency of calcium carbonate in SFE procedures.

### 2.9 Calcium Sulfate (See Supporting Information)

#### 2.9.1 Clinical Recommendations

CaS is a promising biomaterial for bone regeneration due to its biocompatibility, resorption rate, and osteoconductive properties. Limited clinical evidence exists for CaS use in SFEs. While initial clinical outcomes are favorable, particularly in SFE, additional research is needed to solidify its role and optimize its application in clinical practice.

### 2.10 CaP‐Based Ceramics (See Supporting Information)

#### 2.10.1 Clinical Recommendations

CaP‐based ceramics such as BCP and β‐TCP are effective and versatile materials for bone regeneration. Tailoring the HA/TCP ratio in BCP and considering particle size can optimize clinical outcomes. A combination with autologous bone or growth factors to enhance osteoinductive potential can further enhance their efficacy. The costs and uncertainties regarding the effectiveness in SFEs must be addressed.

### 2.11 Hydroxyapatite‐Based Scaffolds (See S1)

#### 2.11.1 Clinical Recommendations

HA comes in a variety of forms, such as beads, porous blocks, and powders. It has shown promising clinical results whether administered alone or in combination with autogenous bone/allografts/xenografts to promote alveolar bone regeneration, transcrestal and lateral SFE.

### 2.12 Application of Autologous Platelet Concentrates

#### 2.12.1 Autologous Platelet Concentrates

In regenerative medicine, autologous platelet concentrates (APCs), either applied alone or in combination with other biomaterials, are widely used bioactive components. The primary biological rationale for utilizing these products is to increase the body's inherent healing ability by concentrating blood‐derived growth factors and cells within the wound microenvironment. The first generation of APC is exemplified by platelet‐rich plasma (PRP). Although various protocols exist for PRP preparation, they generally involve two cycles of centrifugation and have significant limitations due to the use of an anticoagulant initially and an activator afterward, which can interfere with the natural healing process and the release of bioactive molecules. In contrast, the second generation of platelet concentrates is represented by PRF and L‐PRF. L‐PRF preparation involves a single centrifugation cycle and does not require the use of anticoagulants or activators. The process is more cost‐effective and eliminates potential risks associated with the use of activators [74].

##### 2.12.1.1 Transcrestal Approach

The use of bone graft materials in transcrestal SFE has been a source of debate. Therefore, using APCs in this approach when no bone substitutes are used may be a feasible approach. This is mainly due to the acceleration of the healing process and space provision. Also, the membrane forms of APC (such as PRF membrane), can serve as a protective barrier while performing osteotomy as well as closure of perforations [1, 75]. Higher levels of evidence on the application of APCs in transcrestal SFE is scarce. Cho et al. [60], compared PRF membrane and saline as filling materials after hydraulic transcrestal SFE. The 1‐year results revealed a 2.6 ± 1.1 mm versus 1.7 ± 1 mm of vertical bone gain (*p* < 0.05) respectively as well as 100% of implant survival for all implants placed. In another RCT by Lv et al. [76], comparing lateral versus transcrestal SFE using PRF membrane as the sole grafting material, the transcrestal group (initial residual bone height = 3.4 ± 0.8 mm) showed 7.67 ± 1.29 mm of bone height, around 96% of survival rate for implants at 18 months, significantly lower post‐op pain compared with the other group.

##### 2.12.1.2 Lateral Window Approach

A gap in the available evidence with regard to the use of APCs as sole materials in lateral window SFE can be noted. The available evidence mostly relies on prospective case series [77, 78] and retrospective [79, 80, 81] studies. Mazor et al., reported 33 ± 5% of newly formed bone after 6 months in twenty lateral SFEs performed using L‐PRF as the sole graft material. Within the results of all mentioned studies, an average bone height gain ranging from 3.2 ± 1.5 [78] to 10.4 ± 1.2 [67] mm was reported when PRF was used as a sole material in lateral window SFE and simultaneous implant placement. When it comes to implant survival, the studies documented an implant survival rate exceeding 95% across various follow‐up durations. Among them, Simonpieri et al. [67] conducted the longest follow‐up, revealing a 100% implant survival rate over 6 years. Meanwhile, Aoki et al. [80] observed an 85.5% of implant survival over an average follow‐up period of 3.5 years (ranging from 1 to 7 years). Considering studies with inclusion of residual bone height ≥ 4 mm, a cumulative survival rate of 100% was reported, whereas those with residual bone height <4 mm exhibited a rate of 69.6% (*p* = 0.004) [68]. Another important aspect of research on APCs is comparison of their sole use versus bone grafts. An RCT by Merli et al. [82] compared concentrated growth factors (CGF; in membrane form) with DBBM (Bio‐Oss). Twenty lateral SFEs with 1–4 mm of initial bone height, resulted in no difference in implant survival, marginal bone loss and patient satisfaction at the 12 months follow‐up, suggesting comparable outcomes.

Despite several clinical studies on the use of PRF membranes, data on the feasibility of PDGF, PRGF and PRP as the sole grafting material in SFE is still scarce and therefore, no recommendations can be made at this stage [83].

#### 2.12.2 Clinical Recommendations

As APCs release growth factors and have antimicrobial properties, they have been seen as a viable alternative to bone substitutes. L‐PRF has been used in numerous studies due to their physical properties (much stronger than PRP gels or PRGF), autogenous nature, and large release of growth factors over an extended period of time. From authors' experience, we propose that the use of these membranes alone cannot be advised in the case of a limited residual bone height or a two‐stage SFE since they are unable to withstand the pressure within the sinus, which could cause an early collapse or shrinkage. Also, L‐PRF or PRGF membranes have the added benefit of enhancing the repair of the sinus membrane from perforation. They can be used as a barrier membrane to seal off the bony window [84].

### 2.13 Other Graft Substitutes (See Supporting Information)

#### 2.13.1 Summary of Evidence From Latest Systematic Reviews and Meta‐Analyses (2019–2024)

As an evidence‐based approach, we performed a literature search, focusing on assessing the systematic reviews with or without meta‐analysis as well as network meta‐analyses on different SFEs and/or various biomaterials used in these procedures, published within the last 5 years. Table 1 summarizes the findings from these studies.

**TABLE 1:** Summary of the latest evidence (2019–2024) from systematic reviews and meta‐analyses.

Author, Year	Design	Type of SFE	Type of biomaterials	Outcome	Results	Conclusions
Gasparro, 2024 [85]	Umbrella SR	Lateral and crestal SFE	APCs with or without biomaterials.	ISR, IS, IF, postoperative complications, histomorphometric findings, radiographic bone gain, bone volume and bone density.	Positive clinical results, short term new bone formation, and low risk of biological complications when APCs utilized as the only graft material or in conjunction with biomaterials. APCs had no further beneficial effects beyond the physiological healing initiated by the natural blood clot.	Limited evidence on the potential benefits of APCs in long‐term bone regeneration and soft tissue healing.
Khijmatgar, 2023 [86]	NMA	Lateral window SFE	Auto, XG, AG, AP, Bio agents, HA, and combinations of these.	NBF assessed histomorphometrically.	For RBH < 4 mm, XG + AG, XG + AP, and Auto were the highest‐ranked biomaterials. XG + AG performed 8.97 times better compared to other biomaterials. For RBH ≥ 4 mm, Auto, Bio + XG, and XG + auto ranked best.	Performance of materials in terms of NBF may depend on the RBH.
Potdukhe, 2023 [87]	SRMA	Transcrestal using osseodensification and osteotome techniques	NS, 681 participants with 779 implants were included.	Primary implant stability and increase in bone height.	For primary implant stability, the osseodensification group showed higher values than the osteotome group (*I* ^2^ = 20%, *p* < 0.001, pooled mean difference = 10.61 [7.14, 14.08], CI = 95%). For bone height increase, no statistically significant difference observed (*I* ^2^ = 89%, *p* = 0.15, pooled mean difference = 0.30 [−0.11, 0.70], CI = 95%).	Higher primary implant stability with osseodensification technique compared to osteotome technique. No statistically significant difference between groups for mean increase in bone height.
Sivakumar, 2023 [74]	SRMA	NS	411 implants were placed with PRP and 370 implants were placed without PRP3 studies adopted the bilateral split‐mouth design. 3 studies used autografts, and 3 studies employed xenografts.	Cumulative survival and success of dental implants.	Pooled estimate (OR 0.80, 95% CI 0.37 to 1.91; *I* ^2^ = 0%) indicated that there was no statistically significant difference between groups.	Effect of PRP is uncertain on the survival of dental implants in SFE.
Toledano‐Serrabona, 2022 [88]	SRMA	Lateral window	SBS versus AB or DBB.	ISR (%), IF (%), MBL (mm), bone gain (mm), and number of surgical complications. In addition, NFB (%) and residual graft material (%).	Only able to compare BCP with DBB. Greater amount of residual graft material (mean difference [MD]: 4.80 mm; 95% CI: 9.35 to 0.26; P 1/4 0.040) was found in the DBB group.	BCP can be an alternative to DBB in SFE.
Shi, 2022 [89]	SRMA	Transcrestal SFE	421 implants in the graft group and 502 implants in the nongraft group. Bio‐Oss, AB, DBBMs, and β‐TCP in the graft group.	IF rate and MBL.	MA showed no significant difference in the IF rate (RR = 1.03, 95% CI: 1.00, 1.06, *p* = 0.08) or MBL (SMD = 0.06, 95% CI: −0.23, −0.35, *p* = 0.69) between implants with and without graft materials after TSFE. The amount of EBG the non‐grafted group significantly lower than that in the graft group (SMD = −1.07, 95% CI: −1.73: −0.41, *p* = 0.0001).	TSFE in implants with or without grafting can achieve similar results, but there may be more bone gain in TSFE with grafting.
Shah, 2022 [90]	SRMA	Lateral and transcrestal SFE	No graft, PRF, AG, BioOss or ORC graft.	ISR (%).	The overall survival rate for direct approach is 95.98% and for indirect approach is 95.79% with no statistically significant difference.	Technique selected as per the indications given for each direct and indirect procedure.
Canellas, 2021 [35]	NMA	2‐Stage Lateral window SFE	Comparison of six xenograft materials: Bio‐Oss, InduCera, Porous, Osseous, THE Graft and Osteoplant Osteoxenon.	% of NBF % of residual bone substitute measured by histomorphometric analyses from bone biopsies obtained during preparation of the implant site and after 6 months of healing.	After 6 months: Osteoplant Osteoxenon had significantly higher NBF, followed by Bio‐Oss + BMAC, Bio‐Oss + EMD and Bio‐Oss + L‐PRF. In contrast, Bio‐Oss + rhBMP‐2 showed the least NBF.	Osteoplant Osteoxenon produces the most NBF in SFE. L‐PRF, rhBMP‐2, Emdogain did not improve bony healing in SFE filled with Bio‐Oss. BMAC increased the amount of newly formed bone in Bio‐Oss group.
Guo, 2020 [91]	SRMA	Transcrestal SFE	13 non‐grafted TSFE. 5 PC‐TSFE grafted with various platelet concentrations (PRP, PRF, CGF or PRGF).	Implant survival rate (ISR) Marginal/ crestal bone loss (MBL/CBL). Endo‐sinus bone gain (ESBG).	ISR: No significant differences after 1‐year between TSFE (97%, 95%CI = 0.96–0.99) and PC‐TSFE group (99%, 95%CI = 0.97–1.00). MBL/CBL: The 1‐year MBL/CBL value of PC‐TSFE group (0.73 mm, 95%CI = 0.43–1.13 mm) did not show significant difference as compared to TSFE group (0.60 mm, 95%CI = 0.10–1.10 mm). ESBG: no significant enhancement was observed on 1‐year ESBG value on PC‐TSFE group (3.51 mm, 95%CI = 2.31–4.71 mm) in comparison with the TSFE group (2.87 mm, 95%CI = 2.18 m–3.55 mm).	At TSFE sites, grafting platelet concentrations around dental implants did not improve the bone regeneration. Even with restricted RBH, TSFE can be a predictable therapeutic choice for implant sites.
Trimmel, 2020 [92]	SRMA	2‐Stage lateral window SFE	AB (76 SFE), AG (12 SFE), Bovine XG (174 SFE), Porcine XG (8 SFE), BCP (121 SFE), ß‐TCP (106 SFE), Bioactive glass ceramic (Bioglass) (10 SFE), nano HA (26 SFE), nano HA + silica gel (19 SFE), biodegradable copolymer (L‐lactic, D‐Lactic, and glycolic acid) (10 SFE), Bovine + AB 1:1 composite graft (54 SFE), Bovine + AB 1:1 + laser stimulation (6 SFE), Bovine + AB 4:1 composite graft (14 SFE), Bovine + PRF composite graft (19 SFE), Bovine + PRP composite graft (5 SFE), Bovine XG + PRGF composite graft (6 SFE), Bovine + BMA composite graft (6 SFE), Bovine + BMAC composite graft (21 SFE), Bioglass + AB 1:1 composite graft (24 SFE), ß‐TCP + AB 1:1 composite graft (9 SFE), ß‐TCP + PRP composite graft (26 SFE), ß‐TCP + PRF composite graft (8 SFE), AB + PRP composite graft (5 SFE), AB + APC composite graft (12 SFE), BCP + FS composite graft (29 SFE), BCP + PLGA composite graft (16 SFE), BCP + EMD composite graft (10 SFE) and HA + silica gel + PRGF composite graft (10 SFE).	New bone percentage (NB %).	Most effective bone grafting material for NB% was bovine XG + BMC (81%), followed by bovine XG + PRP (77%), bioglass ceramic + AB 1:1 (70%), nano HA + silica gel (70%), and bioglass (70%). AB alone took the 12^th^ position with 57%.	AB alone could not be confirmed as the gold standard for SFE. Composite grafts such as bovine XG + BMC after 5–8 months of healing showed superiority.
Ortega‐Mejia, 2020 [68]	SR	NS	PRF alone or PRF with biomaterials (XG, AG, AP, and titanium‐PRF).	% of NBF, % of residual‐bone substitute material, and % of soft tissue area assessed by histomorphometric analyses. Augmented bone height (mm) was assessed by radiographic evaluation and clinical outcomes in terms of ISQ, IS (%), and postoperative complications.	No additional benefit of PRF in SFE in terms of bone height and % of soft tissue area. Statistically significant lower % of residual bone substitute material in the PRF (+) group compared to the PRF (−) group. % of NBF was slightly higher in the PRF (+) group, but not statistically significant.	No evidence regarding effects of the sole use of platelet concentrates in SFE. Favorable outcomes regarding IS, bone gain, and bone height.
Nino‐Sandoval, 2019 [93]	SRMA	NS	Stem cell group (Bio‐Oss + BMAC system) and the control group (Bio‐Oss).	ISR, increase in bone height, MBL, following implant placement, or NBF.	ISR: not significant when comparing control grafts and grafts with stem cells (*p* = 0.06; Risk ratio (RR) of 4.28, 95% CI 0.95–19.38) Increase in bone height: No difference between the stems cell and control groups (*p* = 0.57; mean difference (MD) 0.38, 95% CI 1.68–0.92). MBL: No significant difference between the groups (*p* = 0. 42; MD 0.27, 95% CI 0.93–0.39). NBF: no significant difference between the stem cells group and the control group (graft without stem cells) (*p* = 0.41; MD 2.21, 95% CI 3.09–7.51).	Use of stem cells did not contribute significantly to greater ISR or the efficacy of bone regeneration following SFE procedure.
Yang, 2019 [16]	SRMA	Lateral and transcrestal SFE	SFE with grafting versus no grafting. Biomaterials used NS.	ISR, MBL EBG, and EBD.	ISR: No statistically significant difference between the two groups, with a RR (risk ratio) of 1.00 (95% CI‐ 0.96–1.04, *p* = 0.94). EBG: more bone gain in the graft group than in the group without graft (95% CI −1.28 to −0.11, *p* = 0.02). MBL: No statistically significant difference between the two groups (95% CI −0.18 to 0.26, *p* = 0.73). EBD: No statistically significant difference between the two groups (95% CI −160.32 to 306.63, *p* = 0.54).	No significant differences between the groups of implants placed with or without grafting in ISR, the changes in peri‐implant MBL and the density of NBF around implants. Bone grafts group showed more EBG than no grafts group.

Abbreviations: AB, autogenous bone; AG, allografts; AP, alloplasts; APC, autologous platelet concentrate; BCP, biphasic calcium phosphate; BMAC, bone marrow aspirate concentrate; DBB, deproteinized bovine bone; EBG, endosinus bone gain; EMD, enamel matrix derivatives; HA, hyaluronic acid; IF, implant failure; IS, implant stability; ISR, implant survival rat; ISR, implant survival rat; L‐PRF, leukocyte‐platelet rich fibrin; MA, meta‐analysis; MBL, marginal bone loss; nano HA, nanocrystalline hydroxyapatite; NBF, new bone formation; NMA, network meta‐analysis; NS, not specified; ORC, oxidized regenerated cellulose; PRGF, plasma rich in growth factors; PRP, platelet‐rich‐protein; RBH, residual bone height; rhBMP, recombinant human bone morphogenetic protein‐2; SBS, synthetic bone substitutes; SFE, sinus floor elevation; SR, systematic review; SRMA, systematic review and meta‐analysis; TSFE, transcrestal sinus floor elevation; XG, xenografts; β‐TCP, β‐tricalcium phosphate.

#### 2.13.2 Overall Clinical Considerations

The presence of 3 mm of residual maxillary bone could allow the insertion of an implant through a lateral window. The new bone formation is also related to the divergence of the sinus walls and from the distance from the medial and the lateral wall: when the angle is lower than 45° and less than 12 mm distance buccolingually the bone regeneration processes are faster, while, in angles wider that 45° and more than 12 mm buccolingually, the formation of new bone could take 1–2 months or more. With 4 mm of residual bone, a lateral window is indicated with simultaneous implant placement. Larger sinuses have been observed to exhibit reduced osteogenic potential due to the increased distance from osteogenic cells in the native bony walls, which may necessitate a longer time for vital bone formation. In a study by Soardi et al. (2011), lateral window SFE surgeries were compared using only allograft in 13 narrow sinuses (with a bucco‐palatal distance of less than 15 mm at 10 mm from the sinus floor) and 15 wide sinuses (with a bucco‐palatal distance of 15 mm or more) [94]. The results showed that at 6‐ and 9‐months post‐surgery, narrow sinuses had a higher percentage of new bone formation—30.5% and 38.8%, respectively—compared with wide sinuses, which had 20.7% and 30.7%. This suggests that wider sinuses may require a longer healing period for the formation of new vital bone.

The presence of  ≥ 5 mm of bone suggests a transcrestal approach, but after confirming that the divergence of the sinus bony walls of less than 45°, it may suggest a more favorable situation with more rapid formation of new bone.

**FIGURE 1:**
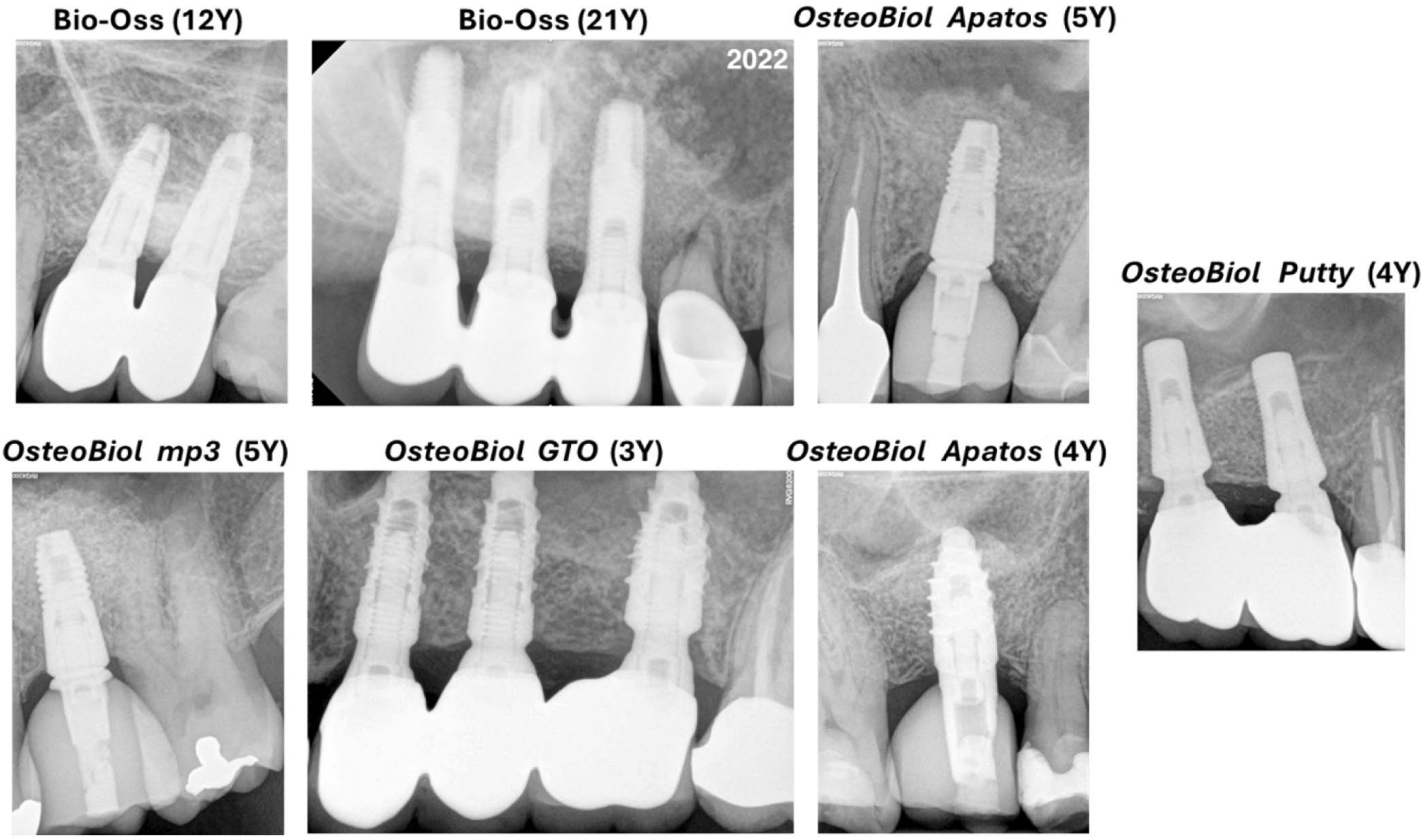
Various biomaterials and their long‐term outcomes appeared in follow‐up x‐rays.

**FIGURE 2:**
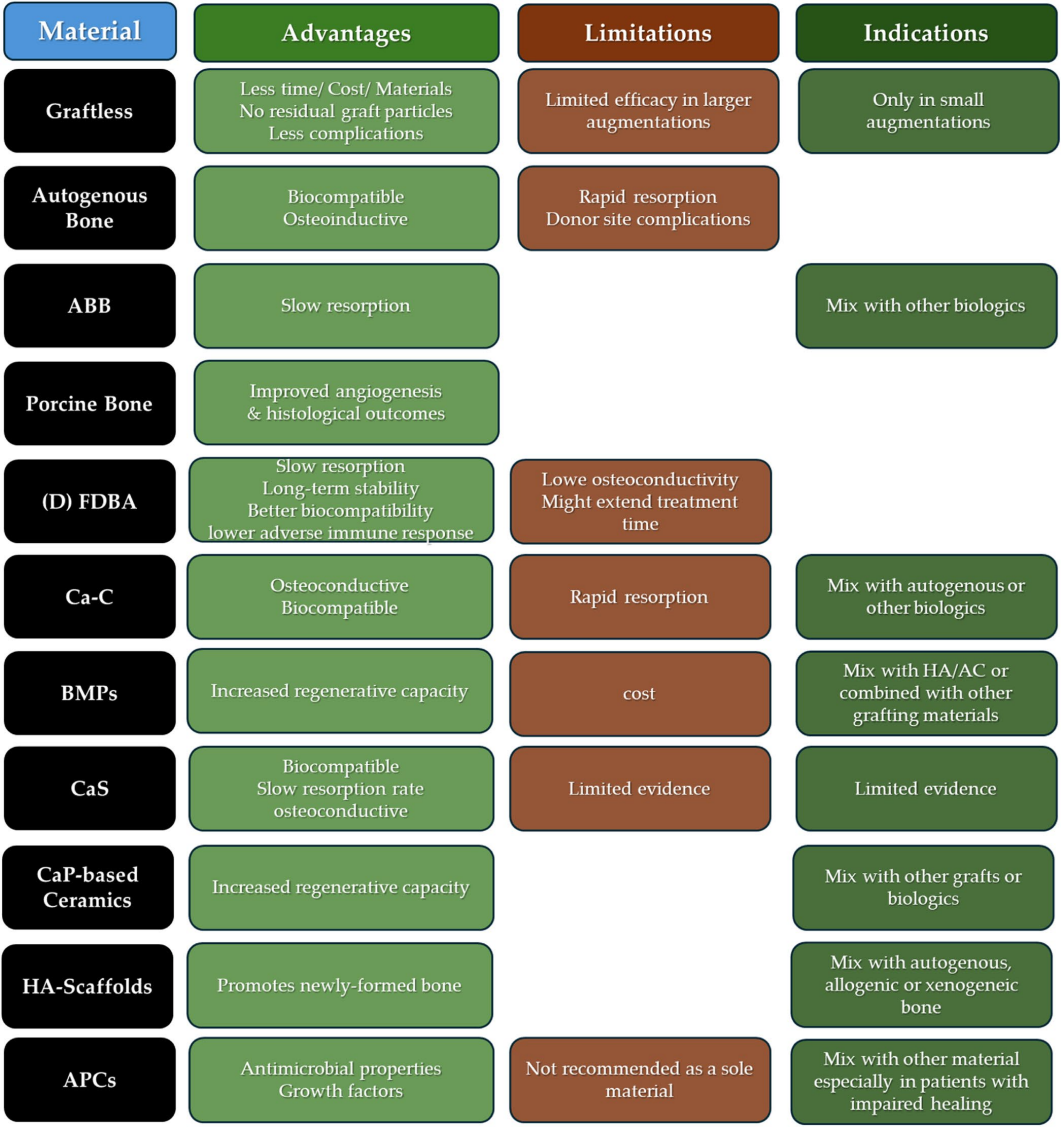
Summary of advantages, limitations as well as key points to consider with regard to each of the reviewed SFE grafting materials.

In a situation when the vertical height of the residual maxillary alveolar crest is ≥ 6, 7 or 8 mm, the approach is crestal. In a lateral approach, the quantity of material needed is usually 1–3 cc. Generally, for granules of higher dimensions, longer is the time required to resorb and substitute the material [70]. Yet, Testori et al. demonstrated through histomorphometric analysis that larger particles of DBBM led to a greater amount of newly formed bone compared with smaller particles [95]. De Molon et al.^102^ showed no statistically significant differences in a histomorphometric analysis in the percentages of biomaterial, newly formed bone, or connective tissue when comparing lateral sinus augmentation using small‐sized versus large‐size DBBM. These differing results could be attributed to variations in patient characteristics, sinus anatomy, surgical techniques, and sample size. Other studies investigating particle size effect on GBR found some advantages for using large particle size [72]. Biomaterials that could be safely used are ABB and porcine xenografts (with this biomaterial, the resorption and substitution by newly formed bone is fast). In the crestal approach, the quantity of needed biomaterial is between 0.2–0.3 and 0.5 ccs, but greater quantities may be necessary for biomaterials with a predictable shrinkage as OsteoBiol Gel 40 [65]. Usually, every 1‐mm of augmentation needs 0.1–0.15 cc. Also, other materials such as Bio‐Oss, DFDBA, FDBA, or OsteoBiol *Apatos* could be used in this indication, but they yield the advantages presented by the more plastic biomaterials. Moreover, the lower resorption rate of these materials must be borne in mind. Figure 1 shows the long‐term outcomes of implants placed in sinus augmentation with various biomaterials. Also, Figure 2 summarizes the advantages, limitations as well as major considerations in the use of various grafting materials used in SFE.

## 3 Conclusions

In conclusion, the presence of residual maxillary bone, ranging from 3 to 8 mm, influences the choice of approach and augmentation procedure for implant placement in the sinus area. The speed of bone regeneration is influenced by factors such as the angle and distance from the sinus walls, with narrower angles and closer distances, as well as the size of lateral window preparation, resulting in faster new bone formation. Biomaterial selection and formulation, such as ABB, and porcine xenografts play a crucial role in achieving optimal outcomes, with considerations for resorption rates and material consistency. Additionally, the use of putty materials offers advantages in terms of handling and sinus membrane elevation. However, it is important to acknowledge the potential differences in resorption rates among various biomaterials.

## Conflicts of Interest

The authors declare no conflicts of interest.

## Supporting information


Data S1.

## Data Availability

The data that support the findings of this study are available from the corresponding author upon reasonable request.
