# Supplementary material for: Clinical Indications and Outcomes of Sinus Floor Augmentation With Bone Substitutes: An Evidence‐Based Review
Source: Clin Implant Dent Relat Res. 2024 Oct 17;27(1):e13400. doi: 10.1111/cid.13400 (PMC11789849; doi:10.1111/cid.13400)
Supplement: Supplementary file 1 — Data S1. [file CID-27-0-s001.docx]

**Supplementary Materials**

**Title:** Clinical indications for different types of graft material and histologic/clinical outcomes of sinus floor augmentation with bone substitutes: An evidence-based approach

**2.7. Bone morphogenic proteins (BMPs)**

Bone morphogenic proteins (BMPs) (recombinant human-derived BMP-2 [rhBMP-2]) are involved in several critical pathways that influence osteoblastogenesis and bone formation^1^. Animal studies^2, 3^ have shown that when is used in combination with carries (absorbable collagen sponge [rhBMP-2/AC], hydroxyapatite [rhBMP-2/HA], tricalcium phosphate etc.), this yields significant amounts of newly bone formation, acceptable implant survival rates and marginal bone loss after functional loading. The efficacy of this material has also been tested in studies on human subjects. Lee et al.,^4^ in a retrospective assessment of 3- and 5-year survival of implants placed after SFE on 45 patients, reported a 100% of survival along with 1.30 ± 0.74 mm of MBL in 5 years in rhBMP-2 group. All outcome variables showed significant improvements compared to the non-rhBMP-2 groups (autografts, allografts or xenografts without rhBMP-2). Similarly, Han et al., compared rhBMP-2/HA versus Bio-Oss (Geistlich Pharma AG, Wohlhausen, Switzerland). After 1 year of loading, similar survival rates and MBL were noted in both groups (90.9% versus 90.5% and 0.65 ± 0.38mm versus 0.85 ± 0.67mm respectively in rhBMP-2 and DBBM).

**2.8. Calcium carbonate**

It is constituted mainly of calcium carbonate (97-98%)^5^, strontium, fluoride, magnesium, sodium and potassium (Biocoral, Morbihan, France). In a comparative study , newly-formed bone was observed bridging the biomaterial particles in select microscopic fields, with no evidence of gaps or connective tissue at the interface when Calcium Carbonate was used in SFEs, although it showed significantly lower new bone formation and higher residual particles compared to phycogene HA^5, 6^. Additionally, the histomorphometric results indicated that some particles displayed signs of resorption, while osteoblasts were actively depositing osteoid matrix directly onto the biomaterial surface. Moreover, fibrovascular tissue, later replaced by newly formed bone, was found within certain pores, and in some instances, grafted particles were in contact with marrow spaces. Notably, no inflammatory infiltrate or foreign body reaction cells were detected throughout the examination.

In a clinical study on 47 lateral window SFEs, using a mixture of aragonitic calcium carbonate and autologous platelet-rich plasma, compared to DBBM and PRP, no significant difference noted between the two groups in terms of histomorphometrical outcomes, microhardness test, and x-ray microanalysis on the newly formed bone^7^. Similarly, Mangano et al.,^8^’s study on 24 SFEs with 68 implants placed, reported following a 6-month healing period, sinuses grafted with calcium carbonate showed a mean vertical bone gain of 6.93 ± 0.23 mm and the histomorphometric analysis revealed 15% ± 3% residual grafted biomaterial, 28% ± 2% newly formed bone, and 57% ± 2% marrow spaces with an implant survival rate of 98.5% in 5 years.

Overall, it can be postulated that this biomaterial is highly osteoconductive, biocompatible, and exhibits significant resorbability^5^ and yields significant potential in successful SFEs either as a sole material or combined with biologic/autologous agents to improve its healing.

**2.9. Calcium sulfate**

Calcium Sulfate (CaS) (Surgiplaster, Classimplant, Rome, Italy) is the oldest biomaterial used in bone regeneration procedures and was introduced in clinical practice at the end of the nineteenth century^9^.

It is a highly biocompatible biomaterial, and it has a relatively fast resorption rate, and in human studies, it tended to be completely resorbed in 6-8 months and completely substituted by newly formed bone^10, 11^. During its resorption, CaS transforms into calcium phosphate, enhancing osteogenic activity, while no inflammatory processes or foreign body reaction cells were observed^12^. Hemihydrated CaS exists in two forms: alpha and beta, with the latter being the most prevalent, typically appearing as powder or granules, and newly formed bone trabeculae tend to envelop areas of pre-existing bone^13, 14^. This newly formed bone presented wide osteocyte lacunae, has a higher affinity for dyes, and, at higher magnification, rims of osteoblasts were observed, depositing osteoid matrix. Many newly formed small blood vessels were found inside the marrow spaces. Only in a few areas, small residues of calcium sulfate were present and were surrounded by newly formed bone^10, 15^.

The CaS biomaterial exhibits high biocompatibility, remarkable resorbability within a few months, and exceptional osteoconductivity^15^. However, clinical evidence and outcomes on the use of this biomaterial in SFEs are still scarce. In a study on 40 SFEs using hydropneumatic elevation technique and injection of a calcium sulfate solution under the antral membrane using a syringe, a sinus membrane elevation of 9.01 ± 3.01 mm and no implant failures were observed at the 1-year follow-up^16^. Other clinical studies have also been conducted though, mostly being case-reports or retrospective studies^17, 18^.

**2.10. CaP-based ceramics**

A meta-analysis investigating augmented maxillary sinuses revealed similar volumes of newly formed bone across various types of biocompatible, osteoconductive bone substitutes, predominantly calcium phosphate (CaP) ceramics^19^. Biphasic Calcium Phosphate (BCP) is an extensively researched synthetic biomaterial, exhibiting similarity in composition and structure to natural bone tissue and possessing commendable osteoconductive properties. BCP consists of two phases: the slowly resorbing Hydroxyapatite (HA) and the more rapidly resorbing TCP. Consequently, the bioactivity and resorption rate of BCP can reportedly be adjusted by altering the HA/TCP ratio. Arunjaroensuk et al.,^20^ tested BCP 70/30 ratio versus DBBM (Cerabone®, Botiss Biomaterials GmbH, Zossen, Germany) in 24 lateral SFEs. Their micro-CT results showed no statistically significant difference in the ratio of bone volume to total volume (BCP 41.51% vs. DBBM 40.97%) and for residual graft material to total volume (BCP 9.97% vs. DBBM 14.41%). Similarly, no significant difference was shown in the histological analysis in terms of bone formation, (BCP 31.43% vs. DBBM was 30.09%) and residual graft area (DBBM 40.76% vs. BCP 45.06%).

In more advanced applications, these synthetic CaP-based bone substitutes have been combined with autologous bone, growth factors, or formulated as fully tissue-engineered cellular constructs^19, 21^ to enhance their osteoinductive potential. These combinations harness the osteoconductive properties of synthetic materials and the osteoinductive capacity of biological components. However, drawbacks such as bone harvesting, or the cost associated with using growth factors persist with this approach. Additionally, the effectiveness of cell-based constructs remains uncertain, as conflicting experimental outcomes have been reported using various cell types of both animal and human origin^22, 23^. Β-TCP is one of the most used synthetic biomaterials for bone reconstruction in oral surgery. It is biocompatible and presents excellent bioresorption and osteoconductive properties^24, 25^. It allows the osteoprogenitor cells to proliferate throughout the bone surface and inside its pores and has been found to induce a low inflammatory response^24, 26^. Comparing β-TCP, bone grafts and no-graft groups in SFE, Loin et al.,^24^ reported that β-TCP induced a local inflammatory reaction similar to a natural bone graft when used in SFE and therefore, can be safely used for sinus augmentation with the advantage of not requiring a donor site and avoiding morbidity. When it comes to the long-term outcomes, a prospective 3- to 9-year follow-up study by Oba et al.,^27^ on 20 transcrestal SFE patients (B-TCP OSferion G1 diameter, 0.5–1.5 mm; Olympus Terumo Biomaterials Corp., Tokyo, Japan), indicated 100% survival rate among the implants, therefore, suggesting clinical success of B-TCP in long-term, nevertheless, the available bone beyond the implant apexes decreased form 3.17 ± 0.97 to − 0.25 ± 1.19 mm. Lastly, concerning the difference in particle dimensions, Mendes et al.,^28^ (30 lateral SFEs) compared autogenous bone, β-TCP ChronOS® (DePuy Synthes®, Paoli, CA, USA) [particle size: 0.5-0.7mm] and BETA-TCP® (Bionnovation®, Biomedical, Sao Paulo, Brazil) [particle size: 0.1-0.5mm]. Following 6 months, the mean bone formation was 51.4 ± 17.4%, 45.5 ± 9.9%, and 35.4 ± 8.0% respectively. The RUNX2 showed higher cellular activity for osteoblast in Group 3 versus 1 and 2. Similarly, VEGF immunolabeling was intense for Group 3 versus moderate in other groups. In conclusion, they suggested the feasibility of B-TCPs as alternative for the autogenous bone graft SFEs.

Steigmann and Garg^29^ compared B-TCP (Cerasorb; Curasan, Kleinostheim, Germany) and PRP in 20 bilateral SFEs (both open and closed), the 6-months’ results indicated more radiographical residual graft materials in B-TCP group. It should be noted that the initial residual bone ridge height was 7-9mm and the study lacked histology outcomes, therefore, restricting the clinical translation of its results. Wiltfang et al., used the same B-TCP material, in combination with PRP to evaluate graft resorption speed. At 6 months of healing, the resorption of β-TCP was not accelerated and foreign-body giant cells and soft tissue surrounding the β-TCP granules were present. While the new bone formation was about 8–10% higher when PRP was used. The results of these studies, overall, suggests that the application of APCs to Ca-P ceramics will only result in accelerated new bone formation if target cells such as osteoblasts or osteocytes are present, and a faster degradation of ceramic bone substitutes cannot be expected.

**2.11. Hydroxyapatite-based scaffolds**

The primary bioceramic material extensively employed for bone grafting in humans is HA, which exhibits a chemical composition and crystalline structure akin to that of bone.

HA and several other calcium-based ceramic materials are categorized as bioactive due to their reported capability to facilitate bone ingrowth. Their bioactivity stems from their osteoconductive properties, fostering the deposition and migration of osteoblasts on the material surface. HA can directly bond with bone. HA, whether used alone or in conjunction with auto/allo/xenografts, has demonstrated favorable clinical outcomes in dentistry and maxillofacial surgery for promoting alveolar bone regeneration. HA is available in various forms, including powders, porous blocks, or beads. In a 6-month histomorphometric clinical trial by Iezzi et al.,^5^ on 30 SFEs using 5 different biomaterials, the results indicated that HA (Algipore®) showed significantly higher newly bone formation compared to calcium carbonate (33.2 ±1.2% vs. 28.1±3.9%). This was similar to other studies such as (Bosshardt et al.,^30^ [lateral SFE, 0.6-mm granules of nanocrystalline HA mixed with the patient's blood; New bone: 28%, residual graft: 25%], Canullo et al.,^31^ [transcrestal SFE, HA-based, sugar cross-linked collagen sponge OSSIX™ Bone; bone height from 5.81 ± 2.20mm to 11.38± 2.50mm at 12-month time-point], Wolf et al.,^32^ [synthetic nanocrystalline nonsintered HA NanoBone™ Artoss; new bone: 21.42% ± 5.70%; residual graft: 39.21 ± 10.03%]).

**2.13. Other graft substitutes**

The utilization of mesenchymal stem cells (MSCs) in maxillary SFE and bone formation has been extensively explored by researchers^33-36^. Studies have demonstrated the efficacy of MSCs derived from various sources, including bone marrow, in promoting bone regeneration. Among these sources, bone marrow-derived MSCs (BM-MSCs) have garnered significant attention due to their abundance and capacity for osteogenic differentiation. BM-MSCs possess the inherent ability to differentiate into osteoblasts, the cells responsible for bone formation, making them particularly suitable for enhancing bone regeneration processes. Additionally, the combination of BM-MSCs with autogenous bone or bone substitutes has been shown to further augment bone regeneration outcomes. Furthermore, recent advancements have explored the potential of adipose-derived stem cells (ADSCs) in maxillary sinus augmentation. ADSCs, obtained from adipose tissue, possess similar osteogenic differentiation capabilities as BM-MSCs and have been investigated for their efficacy in promoting bone regeneration. Studies have demonstrated the proliferative ability and osteogenic differentiation potential of ADSCs, suggesting their potential utility in enhancing bone regeneration in the maxillary sinus region.

**References**

1. Lin GH, Lim G, Chan HL, Giannobile WV, Wang HL. Recombinant human bone morphogenetic protein 2 outcomes for maxillary sinus floor augmentation: a systematic review and meta-analysis. *Clin Oral Implants Res* 2016;27:1349-1359.

2. Susin C, Lee J, Fiorini T, et al. Sinus augmentation using rhBMP-2/ACS in a mini-pig model: Influence of an adjunctive ceramic bone biomaterial. *J Clin Periodontol* 2018;45:1005-1013.

3. Kim CH, Ju MH, Kim BJ. Comparison of recombinant human bone morphogenetic protein-2-infused absorbable collagen sponge, recombinant human bone morphogenetic protein-2-coated tricalcium phosphate, and platelet-rich fibrin-mixed tricalcium phosphate for sinus augmentation in rabbits. *J Dent Sci* 2017;12:205-212.

4. Lee JH, Seo H, Cho YC, Sung IY, Son JH. Addition of recombinant human bone morphogenic protein-2 to the graft materials improves the clinical outcomes of implants placed in grafted maxillary sinus. *J Dent Sci* 2024;19:865-870.

5. Iezzi G, Degidi M, Piattelli A, Mangano C, Scarano A, Shibli JA, Perrotti V. Comparative histological results of different biomaterials used in sinus augmentation procedures: a human study at 6 months. *Clin Oral Implants Res* 2012;23:1369-1376.

6. Piattelli A, Podda G, Scarano A. Clinical and histological results in alveolar ridge enlargement using coralline calcium carbonate. *Biomaterials* 1997;18:623-627.

7. Papa F, Cortese A, Sagliocco R, et al. Outcome of 47 consecutive sinus lift operations using aragonitic calcium carbonate associated with autologous platelet-rich plasma: clinical, histologic, and histomorphometrical evaluations. *J Craniofac Surg* 2009;20:2067-2074.

8. Mangano C, Iaculli F, Piattelli A, Mangano F, Shibli JA, Perrotti V, Iezzi G. Clinical and histologic evaluation of calcium carbonate in sinus augmentation: a case series. *Int J Periodontics Restorative Dent* 2014;34:e43-49.

9. Strocchi R, Orsini G, Iezzi G, Scarano A, Rubini C, Pecora G, Piattelli A. Bone regeneration with calcium sulfate: evidence for increased angiogenesis in rabbits. *J Oral Implantol* 2002;28:273-278.

10. Guarnieri R, Pecora G, Fini M, Aldini NN, Giardino R, Orsini G, Piattelli A. Medical grade calcium sulfate hemihydrate in healing of human extraction sockets: clinical and histological observations at 3 months. *J Periodontol* 2004;75:902-908.

11. Scarano A, Orsini G, Pecora G, Iezzi G, Perrotti V, Piattelli A. Peri-implant bone regeneration with calcium sulfate: a light and transmission electron microscopy case report. *Implant Dent* 2007;16:195-203.

12. Carinci F, Piattelli A, Stabellini G, et al. Calcium sulfate: analysis of MG63 osteoblast-like cell response by means of a microarray technology. *J Biomed Mater Res B Appl Biomater* 2004;71:260-267.

13. Orsini G, Ricci J, Scarano A, Pecora G, Petrone G, Iezzi G, Piattelli A. Bone-defect healing with calcium-sulfate particles and cement: an experimental study in rabbit. *J Biomed Mater Res B Appl Biomater* 2004;68:199-208.

14. Iezzi G, Fiera E, Scarano A, Pecora G, Piattelli A. Histologic evaluation of a provisional implant retrieved from man 7 months after placement in a sinus augmented with calcium sulphate: a case report. *J Oral Implantol* 2007;33:89-95.

15. Pettinicchio M, Sammons R, Caputi S, Piattelli A, Traini T. Bone regeneration in sinus augmentation procedures with calcium sulphate. Microstructure and microanalytical investigations. *Aust Dent J* 2012;57:200-206.

16. Petruzzi M, Ceccarelli R, Testori T, Grassi FR. Sinus floor augmentation with a hydropneumatic technique: a retrospective study in 40 patients. *Int J Periodontics Restorative Dent* 2012;32:205-210.

17. Slater N, Dasmah A, Sennerby L, Hallman M, Piattelli A, Sammons R. Back-scattered electron imaging and elemental microanalysis of retrieved bone tissue following maxillary sinus floor augmentation with calcium sulphate. *Clin Oral Implants Res* 2008;19:814-822.

18. Laino L, Troiano G, Giannatempo G, et al. Sinus Lift Augmentation by Using Calcium Sulphate. A Retrospective 12 Months Radiographic Evaluation Over 25 Treated Italian Patients. *Open Dent J* 2015;9:414-419.

19. Klijn RJ, Meijer GJ, Bronkhorst EM, Jansen JA. A meta-analysis of histomorphometric results and graft healing time of various biomaterials compared to autologous bone used as sinus floor augmentation material in humans. *Tissue Eng Part B Rev* 2010;16:493-507.

20. Arunjaroensuk S, Nampuksa K, Monmaturapoj N, Thunyakitpisal P, Porntaveetus T, Mattheos N, Pimkhaokham A. Gene expression, micro-CT and histomorphometrical analysis of sinus floor augmentation with biphasic calcium phosphate and deproteinized bovine bone mineral: A randomized controlled clinical trial. *Clin Implant Dent Relat Res* 2024;26:402-414.

21. Browaeys H, Bouvry P, De Bruyn H. A literature review on biomaterials in sinus augmentation procedures. *Clin Implant Dent Relat Res* 2007;9:166-177.

22. Meijer GJ, de Bruijn JD, Koole R, van Blitterswijk CA. Cell-based bone tissue engineering. *PLoS medicine* 2007;4:e9.

23. Park JB. Use of bone morphogenetic proteins in sinus augmentation procedure. *J Craniofac Surg* 2009;20:1501-1503.

24. Loin J, Kün-Darbois JD, Guillaume B, Badja S, Libouban H, Chappard D. Maxillary sinus floor elevation using Beta-Tricalcium-Phosphate (beta-TCP) or natural bone: same inflammatory response. *J Mater Sci Mater Med* 2019;30:97.

25. Chappard D, Guillaume B, Mallet R, Pascaretti-Grizon F, Basle MF, Libouban H. Sinus lift augmentation and β-TCP: A microCT and histologic analysis on human bone biopsies. *Micron* 2010;41:321-326.

26. Nyangoga H, Aguado E, Goyenvalle E, Baslé MF, Chappard D. A non-steroidal anti-inflammatory drug (ketoprofen) does not delay β-TCP bone graft healing. *Acta Biomaterialia* 2010;6:3310-3317.

27. Oba Y, Tachikawa N, Munakata M, Okada T, Kasugai S. Evaluation of maxillary sinus floor augmentation with the crestal approach and beta-tricalcium phosphate: a cone-beam computed tomography 3- to 9-year follow-up. *Int J Implant Dent* 2020;6:27.

28. Mendes BC, Pereira RDS, Mourão C, et al. Evaluation of Two Beta-Tricalcium Phosphates with Different Particle Dimensions in Human Maxillary Sinus Floor Elevation: A Prospective, Randomized Clinical Trial. *Materials (Basel)* 2022;15.

29. Steigmann M, Garg AK. A comparative study of bilateral sinus lifts performed with platelet-rich plasma alone versus alloplastic graft material reconstituted with blood. *Implant Dentistry* 2005;14:261-266.

30. Bosshardt DD, Bornstein MM, Carrel JP, Buser D, Bernard JP. Maxillary sinus grafting with a synthetic, nanocrystalline hydroxyapatite-silica gel in humans: histologic and histomorphometric results. *Int J Periodontics Restorative Dent* 2014;34:259-267.

31. Canullo L, Del Fabbro M, Colantonio F, et al. Sinus floor augmentation using crestal approach in conjunction with hydroxyapatite/cross-linked collagen sponge: A pilot study. *Clin Implant Dent Relat Res* 2023;25:974-983.

32. Wolf M, Wurm A, Heinemann F, Gerber T, Reichert C, Jäger A, Götz W. The effect of patient age on bone formation using a fully synthetic nanocrystalline bone augmentation material in maxillary sinus grafting. *Int J Oral Maxillofac Implants* 2014;29:976-983.

33. Gonshor A, McAllister BS, Wallace SS, Prasad H. Histologic and histomorphometric evaluation of an allograft stem cell-based matrix sinus augmentation procedure. *International Journal of Oral & Maxillofacial Implants* 2011;26.

34. Hermund NU, Donatsky O, Nielsen H, Clausen C, Holmstrup P. Long-term changes in graft height after sinus floor augmentation with mesenchymal stem cells in a randomised clinical trial: radiographic evaluation with a minimum follow-up of 2.5 years. *J Dent Med Med Sci* 2013;2:5-14.

35. Rickert D, Sauerbier S, Nagursky H, Menne D, Vissink A, Raghoebar G. Maxillary sinus floor elevation with bovine bone mineral combined with either autogenous bone or autogenous stem cells: a prospective randomized clinical trial. *Clinical oral implants research* 2011;22:251-258.

36. Kaigler D, Avila‐Ortiz G, Travan S, et al. Bone engineering of maxillary sinus bone deficiencies using enriched CD90+ stem cell therapy: a randomized clinical trial. *Journal of Bone and Mineral Research* 2015;30:1206-1216.
